# Clinical and laboratory characteristics of idiopathic inflammatory myositis in Saudi patients: A retrospective study in a single tertiary centre

**DOI:** 10.15537/smj.2023.44.5.20220213

**Published:** 2023-05

**Authors:** Fahidah Alenzi, Jawaher Alotaibi, Manal Alnasser, Fahdah Alokaily

**Affiliations:** *From the Department of Clinical Science (Alenzi), College of Medicine, Princess Nourah bint Abdulrahman University; and from the Division of Rheumatology, Department of Medicine (Alotaibi, Alnasser, Alokaily), Prince Sultan Military Medical City, Riyadh, Kingdom of Saudi Arabia.*

**Keywords:** dermatomyositis, idiopathic inflammatory myositis, muscle weakness, Saudi Arabia

## Abstract

**Objectives:**

Idiopathic inflammatory myositis (IIM) in Saudi patients has been poorly studied owing to the lack of available data. This study aimed to identify the clinical and laboratory features of patients at a single tertiary care center.

**Methods:**

This retrospective study reviewed the medical records of Prince Sultan Military Medical City, Riyadh, Saudi Arabia to collect clinical and laboratory data between December 2022 and February 2017 as follows: age at disease onset, gender, follow-up duration and disease duration; clinical symptoms; laboratory result; presence and type of myositis-specific autoantibody or myositis-associated autoantibody; and type of immunosuppression, presence of malignancy, disease course, and outcome.

**Results:**

There were 26 patients with a mean age of 36.16±13.48, and 84.6% were women. The most prevalent form of IIM was dermatomyositis (n=16, 61.5%), and the most affected organ was the skin. weakness was observed in 25 patients (96.2%), and dysphagia was the most common alarm sign (n=10, 38.5%). During follow-up, the creatine kinase level was elevated at the beginning of the disease and then decreased following treatment, with a mean of 277.73 IU/L. Of the total patients, 17 (68%) were positive for anti-nuclear antibody and 5 (19.2%) were positive for anti-Jo-1.

**Conclusion:**

In our patients, dermatomyositis was the most common form of myositis, and skin manifestations were the most prevalent clinical characteristics. None of the patients developed a malignancy or died.

**I**diopathic inflammatory myopathy (IIM) refers to a group of uncommon systemic disorders that predominantly affect the skin and muscles (characterized by elevation in muscle enzymes, muscle weakness, inflammation on muscle biopsy, and various muscle manifestations), as well as other organs, including the respiratory tract, cardiac system, and gastrointestinal tract.^1,2^ Idiopathic inflammatory myopathy is divided into 4 subtypes depending on the immunohistopathological features, and efficacy of the administered treatment.^1^ The subtypes are polymyositis (PM), dermatomyositis (DM), necrotizing autoimmune myopathy (NAM), and sporadic inclusion body myositis (sIBM). Bohan and Peter created the criteria for diagnosing DM and PM in 1975 by incorporating diagnostic, therapeutic, pathological, and laboratory findings, which remain the standard method in clinical settings.^1^

Phenotypic variations and diagnostic and therapeutic types, predominantly characterized by autoantibodies myositis-specific autoantibody (MSA) and myositis-associated autoantibody (MAA), are present in approximately 70% of myositis patients. In the blood of myositis patients, several autoantibodies can be identified; however, the presence of MSA is not mutually exclusive or distinctive, and represents discrete clinical manifestations.^3^ Patients with myositis and anti-t-RNA synthetase (ARS) antibodies are more likely to experience symptoms, such as fever, arthritis, and Raynaud’s phenomenon.^4^ Similarly, interstitial lung disease (ILD) and amyopathic dermatomyositis have been linked to a group of individuals with anti-MDA5 antibodies.^5^ Furthermore, the prevalence and distribution of MSA, in addition to the clinical manifestations of a specific MSA, may vary depending on the ethnicity and geographical area of the patient.^6^

Several studies have investigated the association between clinical signs, complications, therapeutic responses, and potential treatments in patients with IIM.^7^ For instance, anti-Mi-2 antibodies are associated with mild disease, a good response to treatment, and a fair prognosis, in contrast to anti-MDA5 antibodies, which are linked to lung disease that progresses rapidly and unfavorable outcomes.^1,6-9^ Other studies have also investigated the clinical implications of these autoantibodies.^10^ Ghiradello and Doria hypothesized that MSA correlates with the interaction between muscular and extramuscular mechanisms in patients with IIM. However, there have been no prospective studies investigating the connection between MSA and clinical manifestations, and to date, studies have not been able to definitively determined the pathogenetic, diagnostic, or prognostic role of any of these autoantibodies.

Idiopathic inflammatory myopathy in Caucasian populations has been subjected to various evaluations, and the results revealed that the 5-year mortality rate varies from approximately 52% to 95%.^11,12^ In addition, previous studies have shown that numerous factors can predict poor outcomes, including advanced age, male gender, delay in treatment and diagnosis, anti-Jo-1 positivity, and the presence of dysphonia, cardiac involvement, dysphagia, or respiratory complications.^12-13^ However, diverse studies including different populations have come to varying conclusions, particularly concerning the roles of ethnic groups, clinical characteristics, and type of immunosuppression used. In addition, different detection methods with varying sensitivities and specificities have been studied according to the most up-to-date categorization and evaluation of myositis severity in myositis.^14^

Previously reported survival data mostly reflect the mortality and morbidity rates of non-Chinese patients, and 5 of 22 patients with IIM in the Saudi population were reported in 1993 to have a malignancy.^15,16^ However, to date, there has been no evidence of clinical features, long-term outcomes, or predictive variables in patients with IIM in the Saudi population. This retrospective study investigated the laboratory and patients’ clinical characteristics who were admitted to a single tertiary-care hospital.

## Methods

Patients at Prince Sultan Military Medical City, Riyadh, Saudi Arabia with IIM diagnosed using the 2017 EULAR/ACR classification criteria and who were tested positive for MSA or MAA in the blood with a line immunoblot test were included and reviewed retrospectively between December 2005 and February 2017.^10^ From the records of the IIM patients, the following data were collected: age at disease onset, gender, disease duration, and follow-up duration; clinical symptoms; basic laboratory test results such as renal profile, complete blood count (CBC), albumin, liver function, erythrocyte sedimentation rate (ESR), C-reactive protein (CRP), creatine kinase (CK) level, and serum muscle enzymes; presence and type of MSA or MAA, presence of autoimmune serologic markers such as anti-nuclear antibody (ANA), extractable nuclear antigen (ENA) profile, and association with other rheumatic diseases; and type of immunosuppression administered, presence of malignancy, disease course, and outcome. Pediatric patients and those who did not meet the 2017 EULAR/ACR categorization criteria were excluded.^10^

The Institutional Review Board at Princess Nourah bint Abdulrahman University, Riyadh, Saudi Arabia approved this study (22-0547). Written consent was not required because the data used in this retrospective study were anonymized.

### Statistical analysis

The Statistical Package for Social Sciences, version 26 (IBM Corporation, Armonk, NY, USA) was used to analyze the data after they were entered into an Excel spreadsheet, and descriptive statistics were presented as numbers, percentages, means, and standard deviations (SD). The Chi-square test was used to compare categorical variables. Statistical significance was set at *p*<0.05.

## Results

There were 26 patients in total, of which 16 (61.5%) met the DM criteria, 7 (26.9%) met the PM criteria, 2 (7.7%) met the anti-synthetase criteria, and 1 (3.8%) met the sIBM criteria. The demographic characteristics of patients are shown in Table 1. Women (n=22) constituted a larger group than did men (n=4). The median age of the patients was 43 years (19-77 years), while the median disease duration since onset was 7.0 (1-19) years.

**Table 1:** - Characteristics of the patients’ demographics (N=26).

Characteristic	n	%
***Gender***		
Male	4	15.4
Female	22	84.6
***Ethnicity***		
Saudi	26	100.0
Age (in years), mean±standard	36.16±13.48	
Disease duration, mean±standard deviation	7.18±4.66	
***Type of myositis***		
Dermatomyositis	16	61.5
Polymyositis	7	26.9
Inclusion body myositis	1	3.8
Anti-synthetase syndrome	2	7.7

### Patients clinical and laboratory features

Of 26 patients, 25 (96.2%) had painless muscle weakness (proximal/distal), 9 (56.3%) had Gottron’s papules, 7 (43.8%) had heliotrope rash, 10 (38.5%) had dysphagia, 7 (26.9%) had arthritis, 3 (18.8%) had the V sign, 3 (18.8%) had the Shawl sign, 4 (25%) had mechanics hands, and 2 (12.5%) had Raynaud’s phenomenon. Regarding lung involvement, 9 (69.2%) patients had no lung changes, 1 (7.7%) had no ILD, 2 (15.4%) had mild ILD, and 1 (7.7%) had ILD. Constitutional symptoms, such as malaise was observed in 3 (11.5%), and weight was observed in 1 (3.8%) patient. At diagnosis, the average laboratory test results were as follows: CK, 45.50±20.45; aldolase, 83.89±109.05; ESR, 22.54±20.54; and CRP, 26.85±40.31. Table 2 summarizes the patients’ features.

**Table 2:** - Clinical features of the patients (N=26).

Characteristic	n	%
Painless muscle weakness	25	96.2
Gottron’s papules	9	56.3
Heliotrope rash	7	43.8
Dysphagia	10	38.5
Arthritis	7	26.9
Mechanics hands	4	25.0
V sign	3	18.8
Shawl sign	3	18.8
Raynaud’s phenomenon	2	12.5
Holster sign	0	0.0
Cuticular overgrowth	0	0.0
Periungual erythema	0	0.0
Malignancy	0	0.0
***Lung disease***		
No involvement	9	69.2
Mild interstitial lung disease	2	15.4
No interstitial lung disease	1	7.7
Interstitial lung disease	1	7.7

### Type of auto-antibody

Most of the patients (80.8%) were negative for anti-Jo-1, while 5 (19.2%) were positive. Most patients (68%) were positive for ANA, whereas 32% were negative. Regarding the ANA pattern, 8 (30.8%) had speckled ANA, 7 (26.9%) were negative, and 5 (19.2%) each had nucleolar and homogeneous ANA (Table 3).

**Table 3:** - Frequency for type of autoantibody (N=26).

Type of autoantibody	n	%
***Anti Jo-1***		
Positive	5	19.2
Negative	21	80.8
***Anti-nuclear antibody (ANA)***		
Positive	17	68.0
Negative	8	32.0
***ANA pattern***		
Speckled	8	30.8
Nucleolar	5	19.2
Not available	1	3.8
Negative	7	26.9
Homogeneous	5	19.2

### Association between clinical features and type of autoantibody

Most patients with DM (100%) had gottron papules (*p*<0.05). Two (50%) patients with DM and antisynthetase had mechanics hand (*p*<0.01). Of the patients with DM, 16 (64%) presented with painless proximal muscle weakness (*p*<0.01). These findings suggest an association between Gottron’s papules, mechanics hands, painless muscle weakness (proximal/distal), and IIM types (Table 4). There were no significant associations between other clinical characteristics and IIM types (*p*>0.05).

**Table 3:** - Association between clinical characteristics and idiopathic inflammatory myositis (IIM) types.

Clinical characteristic	IIM types				
	DM	PM	IBM	Anti-synthetase	*P*-value
	n (%)				
Heliotrope rash (Yes)	7 (100.0)	0 (0.0)	0 (0.0)	0 (0.0)	0.112
Gottron’s papules	9 (100.0)	0 (0.0)	0 (0.0)	0 (0.0)	0.035*
V sign	3 (100.0)	0 (0.0)	0 (0.0)	0 (0.0)	0.548
Shawl sign	3 (100.0)	0 (0.0)	0 (0.0)	0 (0.0)	0.548
Raynaud’s phenomenon	1 (50.0)	1 (50.0)	0 (0.0)	0 (0.0)	0.867
mechanics hands	2 (50.0)	0 (0.0)	0 (0.0)	2 (50.0)	0.006**
Painless muscle weakness (proximal/distal)	16 (64.0)	7 (28.0)	1 (4.0)	1 (4.0)	0.006**
Dysphagia	5 (50.0)	4 (40.0)	1 (10.0)	0 (0.0)	0.237
Arthritis	3 (42.9)	2 (28.6)	0 (0.0)	2 (28.6)	0.096

** *p*<0.01,

* *p*<0.05, DM: dermatomyositis, PM: polymyositis, IBM: inclusion body myositis

## Discussion

This study used clinical and laboratory measures to characterize Saudi IIM patients and included 26 patients. The diagnosis of DM was confirmed in 16 of the 26 patients, 7 with PM, 1 with IBM, and 2 with anti-synthetase syndrome. The ratio of male to female patients was 4:22, indicating a female predominance, and was comparable to that of other cohorts.^17^ The mean disease duration was 7.18±4.66 years, and the mean age of onset was 36.16±13.48 years. These results are consistent with those of Srujith et al^18^ who found that the mean age at presentation was 47.3 and Ramesha et al^19^ who found the mean age of 36.5 years.

Clinical findings in the present study included fever (33.3%), painless muscle weakness (96.2%), malaise (16.7%), weight loss (50%), and fatigue (33.3%) as constitutional symptoms, together with cutaneous manifestations (V sign, Shawl sign, heliotrope rash, Gottron’s papules, Raynaud’s phenomenon, and mechanics hands), dysphagia (38.5%), arthritis (26.9%), and respiratory disease (23.1%). These characteristics corresponded with those reported previously.^18-20^ Our patients had a high frequency of DM skin rash, which is consistent with earlier data from Japan. However, this is distinct from Chinese patients, in which the prevalence of DM is lower. This disparity suggests that factors other than autoantibodies may influence the clinical phenotypes of patients with IIM, including environmental and hereditary factors.^21^

Raynaud’s phenomenon had a prevalence of 12.5% in our patients, which is lower than that reported in earlier studies.^18,22^ Moreover, laboratory characteristics included elevated levels of aldolase (n=2), AST, ALT (n=26), and lactic dehydrogenase (LDH) (n=26). ANA test results were positive in 68% of the patients; 8 patients displayed speckled patterns, 5 had homogeneous patterns, and 5 had nucleolar patterns.

Interstitial lung disease was detected in 23.1% of our patients; Douglas et al^23^ found ILD in 18.6% of patients, a lower frequency than that observed in our study. However, Srujith et al^18^ reported a frequency of 37.5% for ILD, which is a notably greater percentage than in our study. Patients with IIM from China appeared to experience IIM-associated ILD more frequently than those from other nations.^24^ Furthser, patients of Chinese, Japanese, and Arab Jordanian descent with IIM were reported to have a higher incidence of ILD than Caucasian patients, and approximately 50% of patients diagnosed with IIM in China and Japan continue to develop ILD, in contrast to only 30% of Caucasian IIM patients.^24-28^

Substantial evidence exists between IIM and cancer. According to several studies conducted in Japan, Europe, and the United States, the IIM’s overall malignancy prevalence ranges from 9 to 40%.^15,29-33^ In contrast, our study revealed a relatively low frequency of malignancy (5.9%), which is comparable with the findings of a study carried out in Arab Jordan.^25^ In the current study, all patients were regularly followed up and underwent screening for over 7 years, and none acquired malignancy or died during this period. In contrast, other researchers have reported the development of a variety of malignancies as well as varying mortality rates.^34-36^

The association between CK levels and IIM outcomes remains unclear. In some studies, CK levels did not achieve any prognostic value for fatality.^37^ However, other studies conducted on the Norwegian cohort by Dobloug et al^38^ and Nuño et al^39^ found patients with DM, PM, and paraneoplastic syndromes had higher total levels of CK. In our study, the muscle enzyme LDH showed the highest elevations. The explanation for this finding is that the overall CK levels were lower because many patients had already been diagnosed and had received immunosuppressive medication before their records were reviewed. In addition, patients who have had IIM for a long time might have near-normal total CK readings; this occurs when a significant portion of damaged muscle is replaced with adipose tissue. Therefore, it is essential to acknowledge that LDH only has a moderate degree of specificity for inflammatory myopathies.^40^

### Study limitation

The current study had some limitations. This was a retrospective study investigating a rare disease, and the number of participants evaluated was limited to a single center. Therefore, future multicenter studies describing patients with IIM are required. To assess organ damage, additional prospective studies are needed, and additional research is needed to validate these findings.

In conclusions, our findings provide compelling evidence that Saudi IIM patients with IIM have favorable outcomes and less damaged organ at long-term monitoring. Further multicenter studies are required to confirm our findings.
